# Editorial: Vascular involvement in eye diseases

**DOI:** 10.3389/fmed.2023.1301145

**Published:** 2024-01-04

**Authors:** Leopold Schmetterer, Doina Gherghel

**Affiliations:** ^1^Singapore Eye Research Institute, Singapore National Eye Centre, Singapore, Singapore; ^2^SERI-NTU Advanced Ocular Engineering (STANCE), Singapore, Singapore; ^3^Academic Clinical Program, Duke-NUS Medical School, Singapore, Singapore; ^4^School of Chemistry, Chemical Engineering and Biotechnology, Nanyang Technological University, Singapore, Singapore; ^5^Department of Clinical Pharmacology, Medical University of Vienna, Vienna, Austria; ^6^Center for Medical Physics and Biomedical Engineering, Medical University of Vienna, Vienna, Austria; ^7^Institute of Molecular and Clinical Ophthalmology Basel, Basel, Switzerland; ^8^Department of Life & Health Sciences, Aston University, Birmingham, United Kingdom

**Keywords:** retinal blood flow, choroidal blood flow, OCT angiography, vascular changes, ocular blood flow

A variety of common eye diseases, including age-related macular degeneration (AMD), diabetic retinopathy (DR), retinal vascular occlusions, glaucoma, and pathological myopia, have a detrimental effect on the ocular blood vessels at many levels, from the anterior to the posterior segment of the eye. As a well-organized ocular vascular system is essential to ensure normal visual function, any damage to these structures may result in loss of vision. Although the current knowledge of the underlying mechanisms for vascular damage in various ocular diseases is quite advanced, more research is necessary to fine-tune our understanding, with the final goal of bringing to our patients better therapies that address this cause of visual morbidities. None of the above would be possible, however, without proper imaging techniques. As such, in parallel with various other studies, more advanced techniques, such as optical coherence tomography angiography (OCTA), have become recently commercially available. This type of technique allows for the visualization of the ocular vasculature *in vivo* with unprecedented resolution, and, as a result, ensures better and earlier diagnosis of ocular vascular disease.

This Research Topic entitled “*Vascular involvement in eye diseases*” brings together 14 articles of various formats, from reviews and original articles, to study protocols and case reports. They highlight various aspects of this field of research, from pathological mechanisms to the use of imaging techniques in the diagnosis of various ocular pathologies. A summary of these articles is presented as follows.

As described above, the OCTA represents an advanced technique for the visualization of the ocular vessels. Although this method allows for the quantification of parameters such as vessel density and foveal avascular zone, no information on volumetric blood flow can be obtained as yet. In their article, Chen R. et al. investigate the OCTA precision in assessing retinal vasculature in children and report moderate-to-good repeatability and reproducibility for papillary and peripapillary perfusion measurement.

In their review article, Tan et al. highlight the importance of ultra-widefield fluorescein angiography and OCTA in the management of retinal vein occlusion, an important sight-threatening vascular disease of the eye. They conclude that the clinical use of these techniques can improve risk stratification and prognostication for neovascularization and cystoid macular edema.

In diabetes, a wide variety of studies has shown that OCTA is capable of identifying microvascular retinal changes before the onset of any clinical features of DR. Li et al. report systemic factors associated with the microvascular alterations including apolipoprotein B and platelets. Hommer et al. investigate retinal neurovascular coupling using Doppler OCT and heart rate variability in patients with type II diabetes. Both parameters were reduced in the patient group as compared to controls but there was no correlation between them.

Idiopathic epiretinal membranes are avascular proliferations between the posterior vitreous cortex and the internal limiting membrane that cause visual impairment including distortion and scotoma. Wang et al. show that choroidal and retinal vascular parameters are associated with both inner and outer retinal morphologic biomarkers in eyes with idiopathic epiretinal membranes.

OCTA is also an invaluable technique to assess the effects of various therapeutic interventions. In a systematic review, Chen X. et al. give an overview of the importance of OCTA in assessing retinal microcirculation in patients who underwent surgery for rhegmatogenous retinal detachment. Even in cases where the retina is fully reattached through primary surgery, the foveal avascular zone area in the deep capillary plexuses was enlarged and vascular density in the foveal deep capillary plexus was reduced. On the same theme, Jiang et al. present a case report that focuses on a pediatric patient with aplastic anemia showing retinal hemorrhages and serous retinal detachment in both eyes and subsequent retinal changes after pars plana vitrectomy.

Another therapeutic intervention in the case of many ocular vascular diseases that result in neovascularisation, is represented by the use of anti-VEGF intravitreal injections. OCTA is, yet again, useful in following up disease evolution in these patients. Türksever et al. showed that age-related macular degeneration subjects treated with anti-VEGF intravitreal injections have reduced OCTA parameters in the retinal vasculature as compared to control subjects, which are, however, not related to the frequency of injections. This confirms that VEGF has vasodilator properties and inhibition of the angiogenic factors induces vasoconstriction.

Glaucoma represents another sight-threatening disease with vascular involvement. Whereas retinal and optic nerve head microvasculature changes have been unequivocally shown in glaucoma, choroidal vascular changes are controversial. Lun et al. did not find any alterations of choriocapillaris microvasculature in patients with primary open-angle glaucoma using optical coherence tomography angiography, which may be expected since the outer retina is largely unaffected in this condition. Differences between retinal vascular parameters were, however, reported in a study by Van Eijgen et al. between primary open-angle glaucoma patients, normal tension glaucoma patients, and healthy controls. This study has also shown that OCTA may not represent a good clinical tool to study vascular dysregulation, because no changes in vascular parameters were observed during provocation tests, such as handgripping.

In addition to the focus on OCTA and related techniques, this Research Topic also presents knowledge on other aspects of vascular involvement in eye disease. Loo et al. review the involvement of the cell surface protein caveolin-1, in vascular abnormalities in glaucoma. The authors emphasize that caveolin-1 is involved in IOP-dependent and independent Mechanisms involving vascular dysregulation. Popa Cherecheanu et al. present two cases with Ehlers–Danlos Syndromes that present with severe vision loss and vascular abnormalities.

Two articles by the group of Benavente-Perez highlight the role of perfusion abnormalities in myopia. A mini-review emphasizes that myopia represents a risk factor for choroidal neovascularisations and glaucoma and indicates the presence of gross, cellular, and molecular alterations at the level of the ocular microvasculature. The same group provides an additional original article that demonstrates how aging interacts with the retinal microvascular changes due to myopia in the marmoset retina.

## Author contributions

LS: Conceptualization, Writing – original draft. DG: Conceptualization, Writing – original draft, Writing – review & editing.

